# Acute lower extremity arterial thrombosis associated with nephrotic syndrome in adults: case series and literature review

**DOI:** 10.1186/s12882-023-03374-0

**Published:** 2023-10-26

**Authors:** Xinqiang Han, Peng Zhao, Zhu Wang, Xingang Ji, Mengpeng Zhao

**Affiliations:** 1 Department of Interventional Medicine and Vascular, Binzhou Medical University Hospital, 256603 Binzhou, Shandong China; 2 Department of Minimally Invasive Interventional Therapy Center, Qingdao Municipal Hospital, 256600 Qingdao, Shandong China

**Keywords:** Nephrotic Syndrome, Thromboembolic events, Arterial Thrombosis, Lower extremity, Adult

## Abstract

**Background:**

Nephrotic syndrome (NS) is a condition associated with hypercoagulability. Thromboembolic events are a well-recognized complication of NS. Venous thrombosis is well known, while arterial thrombosis, which is more severe, occurs less frequently and is mainly reported in children in the literature. The aim of this study was to understand these rare adult cases of NS associated with acute lower extremity arterial thrombosis and draw attention to them to prevent misdiagnosis and delayed treatment.

**Methods:**

From January 2011 and October 2022, we conducted a retrospective study of patients with NS and arterial thrombosis. Their clinical manifestations, imaging characteristics, treatments and outcomes were analyzed and compared, and a literature review was performed.

**Results:**

Nine adults with NS and acute lower limb arterial thrombosis were described. In seven of these patients, six had fresh thrombi that preceded the NS diagnosis, while one had a history of NS for 14 years and previously underwent an emergency thrombectomy. Three of the seven patients eventually underwent above-knee amputations, and the other four underwent arterial revascularization with satisfactory recovery of lower-extremity perfusion. In addition to the seven patients mentioned above, the other two received successful anticoagulant treatment, as the thrombosis was present only in the popliteal artery.

**Conclusion:**

Acute lower extremity arterial thrombosis is a rare but serious and potentially lethal complication in patients with NS, and early recognition and appropriate management are crucial for good patient outcomes.

## Background

Thromboembolic events have been established as one of the most serious clinical complications in adults with nephrotic syndrome (NS); they often lead to severe consequences such as limb loss and are potentially life-threatening. These complications in NS are multifactorial. The hypercoagulability of NS is one of the leading causes of the increased thrombotic tendency [1, 2].

Urinary loss of anticoagulants, thrombocytosis, increased synthesis of procoagulants and increased blood viscosity have been noted in nephrotic patients. All these alterations could result in the development of a hypercoagulable state [2, 3]. Among all the potential mechanisms, excessive excretion of anticoagulants, especially antithrombin, and enhanced platelet aggregation are considered to be the most important contributing factors to thromboembolic events [4].

Venous thrombosis is a well-known complication, while arterial thrombosis, which is extremely rare, is more severe and has mainly been reported in children with NS [5]. In some cases, arterial thrombosis was the initial clinical manifestation, leading to the subsequent discovery of NS [6, 7]. To our knowledge, at present, no more than 22 cases of spontaneous lower extremity arterial thrombosis in adults with NS have been reported in the English literature [8–12], and the majority of these were single cases [10, 13–15]. In this study, we report nine adults who presented with NS and acute lower extremity arterial thrombosis and summarize the clinical and prognostic characteristics of such thromboembolic events of NS more comprehensively than in previous reports, with the purpose of understanding this rare complication and preventing misdiagnoses.

## Methods

We conducted a single-center retrospective study and searched the electronic medical records system at our institution using the following keywords: “Nephrotic syndrome”, “Arterial thrombosis”, and “Lower extremity” between January 2011 and October 2022.

The inclusion and exclusion criteria were as follows: (1) Inclusion criteria: ① definite diagnosis of nephrotic syndrome; ② lower extremity arterial thrombosis; ③ acute onset; ④ no history of diabetes or arteriosclerosis obliterans of lower extremities. (2) Exclusion criteria: ① severe abnormalities of the heart, liver, kidney, and blood coagulation that render surgical treatment impossible; ②previous diagnosis of autoimmune/immune-mediated diseases and cancer.

The electronic medical records (clinical manifestations, management and outcome), laboratory examination results(blood routine, coagulation analysis, D-dimer, five items of blood lipids, liver and kidney function, blood glucose and muscle enzyme profile)and imaging data (ultrasonography, computed tomography angiography (CTA) and digital subtraction angiography (DSA) findings) of these patients were collected and analyzed retrospectively.

The hospital ethics committee approved the study and the inclusion of as many cases as possible (No. LW-23).

## Results

### Baseline characteristics

Nine patients with NS and arterial thrombosis, eight adult males and one adult female, were ultimately included in the study. Their median age was 43.1, with a range of 23 to 67 years. All but one patient had no history of ischemic arterial diseases. All nine adults presented with unilateral acute lower limb thrombosis, and the median onset time was 2.8 days. One patient with a 14-year history of minimal change nephropathy and six with an unremarkable previous medical history, including no drug use, no smoking history, and no cardiac disease or diabetes, were admitted to the emergency department for acute severe pain and numbness of the lower extremities. Two other patients had a history of primary focal segmental glomerulosclerosis for 3 and 6 years; they were being treated at the time with prednisolone and were admitted from the outpatient clinic for sudden intermittent claudication that was present for no longer than one week. All patients had no prior diagnosis of autoimmune/immune-mediated diseases, cancer, lower extremity arteriosclerosis obliterans or diabetes. The clinical details of the patients are provided in Table 1.

**Table 1:** The clinical characteristics at baseline and outcomes

Case series	Gender	Gender	*Time (months)	Serum albumin (g/L)	Urinary protein (g/24 h)	D-dimer (mg/L)	Fibrinogen (g/L)	Arterial thrombus localization	Amputation	Diabetes mellitus	Hypertension	Dyslipidemia	Smoke
No.1	Male	26	5	15.2	26.5	7.86	7.99	Iliac	Yes	No	No	Yes	Yes
No.2	Female	33	4	24.2	7.84	12.5	6.97	Iliac-femoral- popliteal	Yes	No	No	Yes	No
No.3	Male	52	5	15.6	10.65	13.69	5.39	Femoral-popliteal	No	No	Yes	Yes	Yes
No.4	Male	50	7	20.5	8.21	3.59	5.59	Popliteal	Yes	No	No	Yes	Yes
No.5	Male	23	6	14.8	5.55	12.13	6.20	Popliteal	No	No	Yes	Yes	No
No.6	Male	42	9	18.3	22.53	17.17	6.86	Popliteal	No	No	Yes	Yes	Yes
No.7	Male	39	13	22.9	6.3	2.56	4.25	Femoral	No	No	Yes	Yes	No
No.8	Male	67	8	21.0	11.08	5.07	6.10	Iliac	No	No	Yes	Yes	Yes
No.9	Male	56	7	19.95	17.35	4.38	5.72	Femoral-popliteal	No	No	Yes	Yes	Yes

* Time from clinical onset to clinical remission of nephrotic syndrome

### Diagnosis and treatment process

**Fig. 1:**
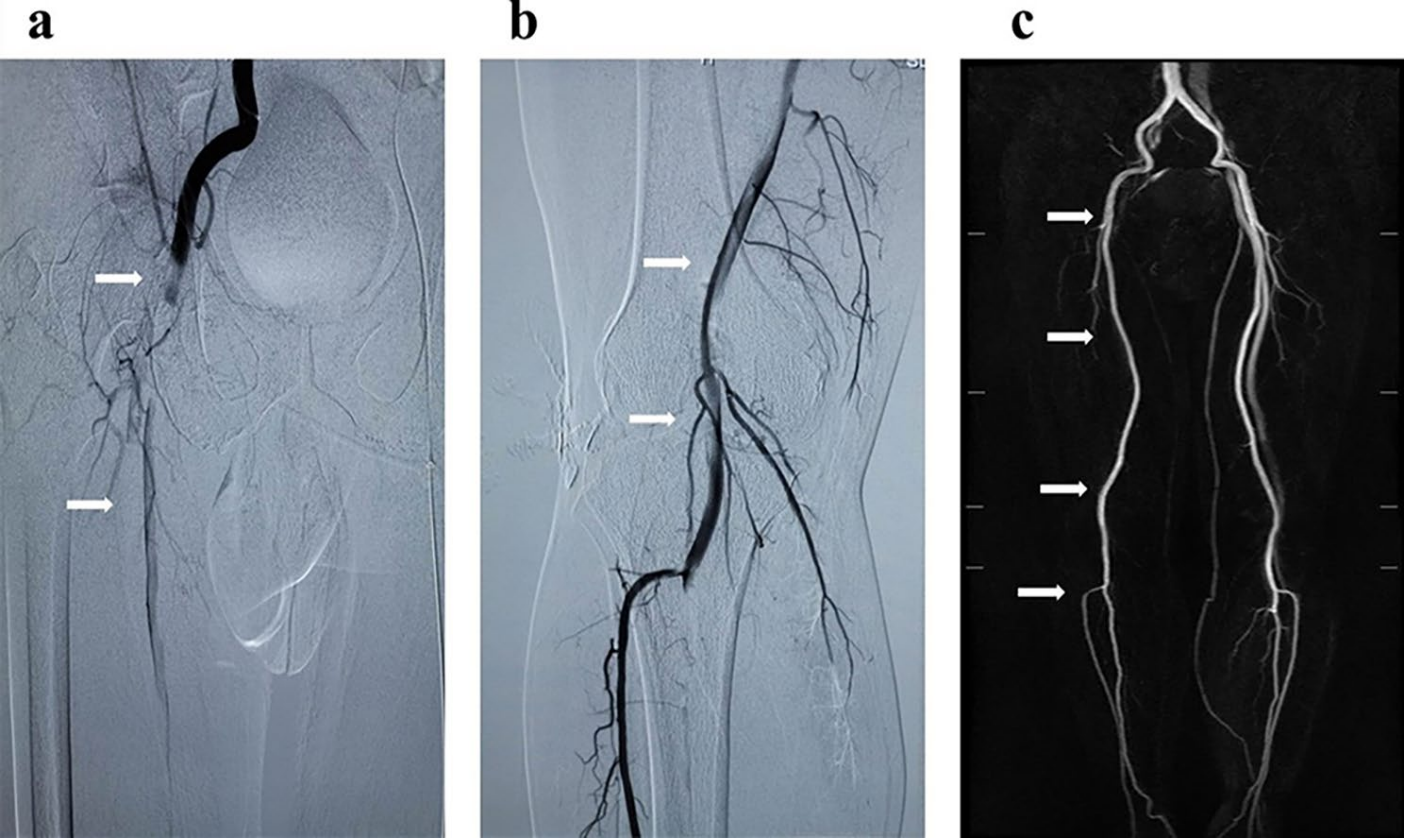
Arteriogram of the right lower extremity showed a complete occlusion with a filling defect of the femoral artery (**a**, arrows) and popliteal artery (**b**, arrows), and MRI without contrast agent (**c**, arrows) showed disappearance of the right femoral-tibial thrombosis after thrombectomy and thrombolysis

Physical examination of the six patients admitted to the emergency department showed bilateral lower extremity pitting edema with varying degrees. Urgent CTA or ultrasonography revealed lower limb arterial thrombosis. Emergency intraarterial thrombectomy with the AngioJet system and/or catheter-directed thrombolysis were performed for limb salvage (Fig. 1). All patients received preoperative and postoperative anticoagulation with low molecular weight heparin (LMWH) at a dosage of 1 mg per kg of body weight twice daily and anti-platelet aggregation with Aspirin at a dosage of 100 mg one nightly and thrombolysis was performed using urokinase (400,000-600,000 units per day) until the previous visible thrombus completely disappeared or fibrinogen decreases below 1.0 g/L, as determined by the assessment of the angiogram.

Laboratory investigations showed decreased serum albumin levels (< 25 g/L) and elevated serum cholesterol and triglyceride levels. Urinalysis revealed positivity for urine proteins. The 24-hour urine protein excretion was more than 3.5 g. Biopsies were not taken of the six undiagnosed patients owing to concerns of possible kidney hemorrhage, while anticoagulants and antiplatelet agents could not be discontinued because of arterial thrombosis. Based on the clinical symptoms and laboratory findings, the eventual diagnosis of NS was established by a multidisciplinary expert team including nephrologists. Thereafter, corticosteroid therapy was initiated for these six patients, and the other patient with minimal change nephropathy received an adjusted prednisone dosage of 50 mg per day.

**Fig. 2:**
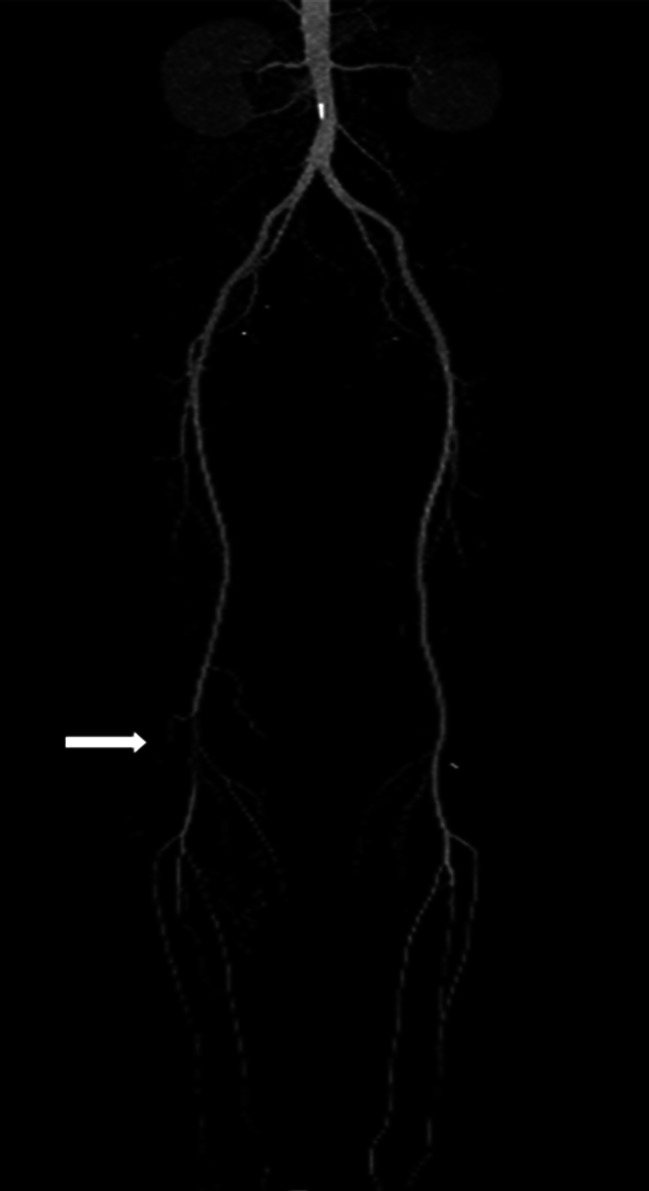
CTA showed a filling defect (arrow) in the popliteal artery due to thrombus formation

In addition to the seven patients mentioned above, the other two were diagnosed with NS and had been treated with prednisolone at a dosage of 50 mg/day for eight weeks, which was tapered off gradually to 10 or 20 mg/day. CTA or ultrasonography revealed only popliteal artery occlusion, but the trifurcation arteries were still visible in 2 patients (Fig. 2). Laboratory data showed that serum albumin decreased to less than 20 g/L. Their 24-hour urine protein excretion exceeded 5 g/day. These patients underwent anti-coagulation with LMWH (1 mg/kg body weight twice a day subcutaneously) and anti-platelet aggregation with Aspirin (100 mg one nightly) as soon as they were diagnosed with arterial thrombosis. Meanwhile, their prednisone dosage was adjusted to 50 mg per day, and tacrolimus or cyclophosphamide was added. Trace bilateral lower-extremity mild pitting edema was present in both patients.

### Outcome

**Fig. 3:**
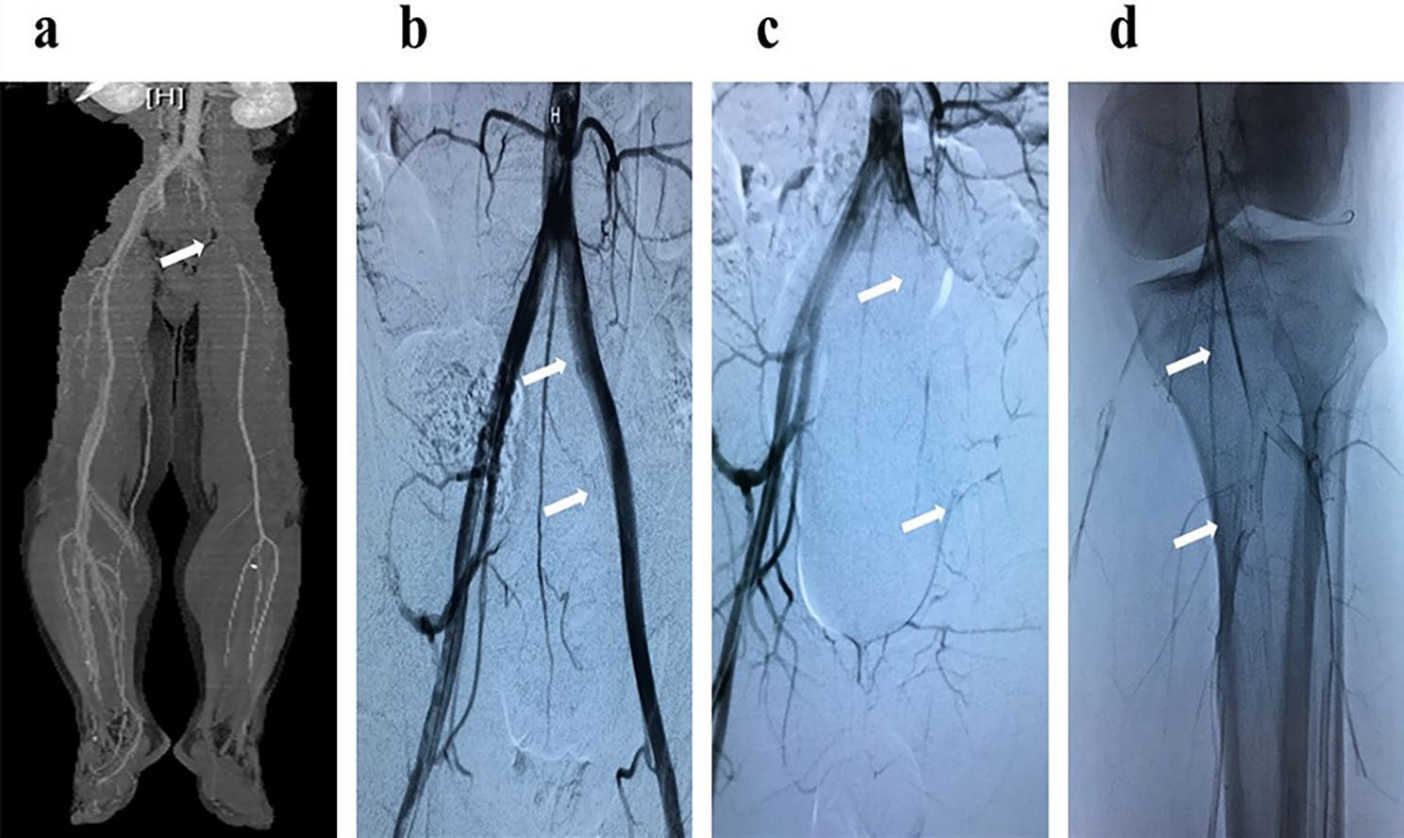
CTA showed (**a**) occlusion of the left iliac artery (arrow) and without grossly visible atherosclerotic lesions, arteriogram showed (**b**) complete recanalization (arrows) of the iliac artery after thrombectomy, and the second arteriogram showed complete occlusion (**c**) of the iliac and femoral vessels (arrows) and a filling defect (**d**) in the below-the-knee arteries (arrows)

After percutaneous mechanical thrombectomy (PMT) and local thrombolysis, good distal arterial flow was reestablished in six patients, and the symptoms of lower limb ischemia completely resolved within 24 h. However, the other patient had poor run-off distal to the popliteal artery. Also, two of the six patients suffered symptomatic recurrence on the third and seventh postoperative days, despite the same anticoagulant and anti-platelet aggregation therapy as the others. Reexamination angiography revealed fresh thrombus recurrence in the previously affected arteries and in the distal femoral and popliteal arteries (Fig. 3). The ischemia of the lower extremities worsened despite surgical thrombectomy and thrombolysis, and unfortunately, these three patients had to undergo major amputation of the lower extremities. All patients were discharged with oral warfarin or rivaroxaban and prednisolone. According to the available follow-up data in the electronic medical records system, none of the seven patients experienced arterial thrombosis recurrence.

Arterial flow was re-established with satisfactory recovery of lower extremity perfusion and function in the other two patients after one month of anticoagulation and anti-platelet aggregation without surgery. Additionally, steroid therapy was continued with an adjusted regimen after consultation with a nephrologist. Four months later, the kidney function of both patients had also recovered. The serum albumin level increased to more than 30 g/L, and the 24-hour urine protein excretion decreased to below 0.5 g/day. At the last visit, no recurrence of thromboembolic complications had occurred. Ultrasound of the popliteal arteries demonstrated partial vessel recanalization at a post-discharge follow-up of half a year.

## Discussion

This study demonstrates the unfavorable outcome of nephrotic syndrome patients presenting with acute lower extremity arterial thrombosis. Three patients underwent unavoidable lower limb amputations, like what has been previously reported [7–9, 15]. In contrast, the outcome was slightly better when arterial thrombosis occurred during glucocorticoid therapy [11, 14], although some scholars have argued that steroid therapy can induce a state of hypercoagulability and provoke thrombosis [13].

To the best of our knowledge, patients with NS are in a hypercoagulable state with an increased risk of developing thromboembolic events [16]. Affected individuals are typically prone to developing venous system thrombosis, especially in the lower limbs and renal veins. Additionally, the very rare occurrence of arterial thrombosis is predominantly observed in children, and there are only a limited number of case reports in the literature [7, 11, 12]. Despite the lower incidence, in previous studies, researchers found that the isolated femoral artery appears to be the most susceptible, which may be closely related to artery injury during femoral vein puncture. [8] However, Kim et al. found no relationship between arterial thrombosis and vein puncture [17]. Similarly, in our four patients with femoral artery involvement, no relevant vein puncture history was found.

The pathophysiology of thrombosis in NS is multifactorial and has been extensively reported in the literature [4, 18]. However, the mechanism of hypercoagulability has not yet been fully elucidated. It is speculated that lower concentrations of anticoagulant proteins and serum albumin (particularly < 20 g/L) are obvious risk factors for arterial thrombosis secondary to NS [2]. In the present study, all six patients had serum albumin levels lower than 25 g/L, which also supported the findings of previous studies [18]. However, arterial thrombosis still occurs in some NS patients with serum albumin levels greater than 30 g/L [19]. Apart from the abovementioned characteristics, one of the outstanding features of NS is hyperlipidemia. Patel et al. found that long-term steroid therapy and/or hyperlipidemia could cause chronic NS patients to stay in a microscopic atherosclerotic state [20]. Traditionally, macroscopic atherosclerosis is a direct restriction of blood flow secondary to thrombosis. However, Hoak et al. found that microscopic atherosclerosis was also able to impair endothelial function and promote thrombosis [21]. This may be evidence of spontaneous arterial thrombosis in the nine patients in this study, although none of them had macroscopic atherosclerosis or significant stenoses.

Fibrinogen is a critical coagulation factor that can cause endothelial dysfunction and platelet aggregation and plays a pivotal role in atherosclerosis initiation and development [22]. An increase in fibrinogen levels generates a larger amount of fibrin cleavage fragments, which in turn generate denser and insoluble fibrin networks and may contribute to hypercoagulability, thus promoting platelet hyperaggregability in patients with NS [23]. D-dimers are generated by fibrinolysis of fibrinogen and fibrin [24]. The D-dimer level has been suggested to be an indicator of hypercoagulability and hyperfibrinolysis and is an excellent predictor of near-term thromboembolic events [25]. In the present study, both the fibrinogen and D-dimer levels increased to varying degrees, and the elevations were obvious in all patients, which indicates ongoing thrombogenic and fibrinolytic activities.

Regarding sex, male patients accounted for the most of the cases, which was consistent with previous studies [26]. Currently, it is not clear why such a sex-based difference exists. Arterial thrombi are mainly composed of platelets (white clots), and the prevalence of dysfunctional platelets was significantly higher in males than females, which might be one of the reasons [27, 28].

In addition, thrombosis is most frequent during the initial months, especially half a year after biopsy, regardless of histological subtype, and it can also occur at any stage after the onset of NS [26, 29]. According to the literature, iliac-femoral artery thrombosis has rarely been reported in adults with NS, with a high degree of limb amputation and a very poor prognosis [17]. In our two cases involving iliac arterial thrombosis, the patients finally underwent major amputation despite the initial revascularization being very good. It is critical to recognize that once acute arterial thrombosis occurs, it progresses and deteriorates rapidly. Prompt diagnostic workup and treatment are crucial to salvage life and limbs. However, no consensus has been reached concerning treatment protocols in this situation, which depend on the location of the thrombosis and hypercoagulable state [30]. Nevertheless, the rapid recanalization of arterial occlusion and keeping the ischemia time as short as possible are of the utmost importance [31]. PMT is one of the choices for early thrombus removal that can rapidly relieve ischemic symptoms and prevent disease progression, especially when a greater thrombotic burden is present, as it may facilitate more complete removal of the thrombus and revascularization compared to manual thrombectomy [32]. However, we must acknowledge the potential impairment of renal function as a result of intravascular hemolysis caused by rheolytic PMT with the AngioJet system. For this reason, we adopted a series of interventions, including adequate perioperative hydration, urine alkalization, diuresis and close monitoring of renal function. Additionally, magnetic resonance perfusion imaging without contrast media was also used as a means of assessing vessel patency. None of the four patients who underwent mechanical thrombectomy developed severe kidney injury.

Although the patients had an excellent distal outflow tract after thrombectomy and thrombolysis and received a standard combination of anticoagulation and antiplatelet therapy, the thrombosis rapidly recurred, leading to major amputation. Untreated NS, the predisposing factor for thrombosis, has not yet been excluded in this condition and may have the potential to contribute to the development of thrombosis in similar cases, possibly as the primary reason for the modest efficacy of antithrombotic and thrombolysis therapy. However, the agents used for the management of NS have a slower onset of action, and their preoperative use makes little sense for patients undergoing emergency surgery [33]. Despite consideration of the diagnosis of NS and the prompt administration of corticosteroids, the thrombosis recurred in the two patients with arterial thrombosis as the initial manifestation. In contrast, the application of corticosteroids prior to elective surgery or adjustment of the dosage of corticosteroids in combination with cytotoxic agents for patients with NS recurrence can improve hypoalbuminemia and proteinuria due to NS [34]. The other four patients had no further thrombosis relapses after controlling for the primary NS. Therefore, attention should be given to the treatment of NS to save the ischemic limbs.

There is no consensus on whether patients with NS should be treated with anticoagulation or antiplatelet therapy at present. Anticoagulant therapy is the process of reducing the endogenous and exogenous coagulation pathways through the application of drugs, and the process of reducing fibrin thrombosis. Antiplatelet is the process of taking anti-platelet aggregation drugs to reduce the adhesion and aggregation function of platelets, thereby reducing the formation of platelets and thrombosis. Hypercoagulability and enhanced platelet aggregation is considered to be the most important factors leading to lower extremity arterial thrombosis. The author thinks that anticoagulation and antiplatelet therapy are equally important for NS and arterial thrombosis because combined antithrombotic therapy maybe promote recanalization of blood vessel and prevent recurrence of thrombosis.

In addition to the typical clinical manifestations, it is necessary to select appropriate work-up to make a definite diagnosis of NS. Renal biopsy is essential for the diagnosis of NS if the patient has no contraindications. However, renal biopsy is often contraindicated in patients with NS and lower extremity arterial thrombosis. We can perform laboratory examinations such as ANA, anti-dsDNA, ANCA, anti-MPO and anti-PR3 [35], cryoglobulins, C3 and C4, serum and urinary immunofixation, free light chains [36], anti-THSD7A, anti-PLA2R [37] to exclude autoimmune and immune-mediated diseases, cancer and other diseases leading to hypercoagulable state [38], so as to enhance the reliability of the diagnosis of NS.

This study also has certain limitations, such as monocentric retrospective study, little cohort, absence of important diagnostic laboratory parameters. In the future, we will conduct a prospective multi-center further study.

## Conclusion

Lower extremity arterial thrombosis has an acute onset and obvious symptoms. The outcome of NS complicated with arterial thrombosis was unsatisfactory due to the high rates of limb loss. Early diagnosis and prompt treatment of arterial thrombosis secondary to NS and primary nephrotic disease are of the utmost importance for patient outcome.

## Data Availability

The datasets used or analyzed during the current study are available from the corresponding author on reasonable request.

## Abbreviations

***NS***: Nephrotic Syndrome
***CTA***: Computed Tomography Angiography
***DSA***: Digital Subtraction Angiography
***LMWH***: Low Molecular Weight Heparin
***PMT***: Percutaneous Mechanical Thrombectomy
